# Preoperative Advanced Lung Cancer Inflammation Index as a Potential Marker for Incidental Gallbladder Carcinoma: A Matched Case–Control Study

**DOI:** 10.3390/medicina62020269

**Published:** 2026-01-27

**Authors:** Yusuf Yunus Korkmaz, Oguzhan Aydin, Huseyin Karatay, Mehmet Saban Korkmaz, Sadik Peker, Feyyaz Gungor, Erdem Kinaci

**Affiliations:** 1 Department of General Surgery, Istanbul Başakşehir Çam and Sakura City Hospital, Istanbul 34480, Turkey; droguzhanaydin@gmail.com (O.A.); korkmazmehmetsaban@gmail.com (M.S.K.); sadkpeker34@gmail.com (S.P.); feyyazgngr@gmail.com (F.G.); erdemkinaci@gmail.com (E.K.); 2 Department of Pathology, University of Health Sciences, Başakşehir Çam and Sakura City Hospital, Istanbul 34480, Turkey; huseyin140@gmail.com

**Keywords:** gallbladder neoplasms, cholecystectomy, inflammation, biomarkers, risk assessment, retrospective studies

## Abstract

*Background and Objectives:* Incidental gallbladder carcinoma (IGBC) is an uncommon but clinically significant finding after elective cholecystectomy, as failure to recognize malignancy preoperatively may lead to inadequate initial surgical management. Inflammation-based hematological indices have been explored in various gastrointestinal malignancies; however, data focusing on preoperative discrimination of IGBC are limited. This study aimed to evaluate the diagnostic performance of the Advanced Lung Cancer Inflammation Index (ALI) and other inflammatory and immunonutritional indices in distinguishing IGBC from benign gallbladder disease. *Materials and Methods:* This retrospective matched case–control study included patients who underwent elective cholecystectomy between 2020 and 2025. Nineteen patients with histopathologically confirmed IGBC were matched 1:4 by age and sex with 76 patients with benign gallbladder disease. Preoperative laboratory parameters obtained within 72 h before surgery were used to calculate inflammatory indices, including NLR, PLR, LMR, SII, CAR, ALI, and the CALLY index. Diagnostic performance was assessed using receiver operating characteristic (ROC) analysis. Independent associations with malignancy were evaluated using matched conditional logistic regression. *Results:* No significant differences were observed between benign and malignant groups in baseline demographic or routine laboratory parameters. Among derived indices, ALI and LMR demonstrated borderline differences, representing the closest discriminatory markers. In ROC analysis, ALI showed the highest diagnostic performance (AUC = 0.69), followed by LMR (AUC = 0.639). In matched conditional logistic regression, ALI was independently and inversely associated with malignancy (adjusted OR = 0.997 per unit, 95% CI: 0.9937–1.0000; *p* = 0.04); when rescaled per 100-unit increase, the adjusted OR was 0.93, whereas LMR did not reach statistical significance. *Conclusions:* Among evaluated preoperative inflammatory and immunonutritional indices, ALI demonstrated a more consistent association with incidental gallbladder carcinoma. Although its discriminative ability was moderate, ALI may serve as a complementary biomarker for preoperative risk stratification when integrated with clinical and radiological assessment.

## 1. Introduction

Gallbladder cancer (GBC) is a rare gastrointestinal malignancy with an aggressive clinical course and high mortality [1,2,3]. Because of the generally asymptomatic presentation and overlapping symptoms with chronic calcific cholecystitis, preoperative diagnosis is difficult in most patients, resulting in an incidental diagnosis of GBC in about 0.2–2% of patients who undergo elective cholecystectomy [1,2]. As surgical resection represents the only curative treatment for GBC, omission of radical cholecystectomy—including hepatic resection of segments IVb–V and regional lymphadenectomy—at the initial operation significantly compromises survival outcomes [3]. Moreover, incidental GBC patients who undergo reoperation due to the residual tumor generally face greater complication-related morbidity and mortality due to the increased difficulty of reoperation because of postoperative fibrosis and adhesions, with associated delays in accurate tumor staging [3,4].

Chronic inflammation, immune response dysfunction, and nutritional status play important roles in the biology of GBC. Accordingly, inflammation-based hematological indices have been investigated in many solid tumors [5]. There is evidence that indices such as the neutrophil/lymphocyte ratio (NLR), platelet/lymphocyte ratio (PLR), lymphocyte/monocyte ratio (LMR), and CRP/albumin ratio (CAR) have prognostic effects in biliary system malignancies; however, the results are inconsistent, and data specific to incidental GBC are quite limited.

The Advanced Lung Cancer Inflammation Index (ALI) is a composite biomarker that combines body mass index, serum albumin level, and neutrophil/lymphocyte ratio to reflect both systemic inflammation and the immunonutritional status. First defined in lung cancer, ALI has been reported to outperform individual inflammation indices in predicting survival in various gastrointestinal malignancies in recent years. Song et al. demonstrated that ALI is one of the strongest prognostic markers in lung cancer patients, while Ma et al. reported that low ALI levels are associated with worse postoperative outcomes in operable cases [6,7].

Similarly, in hepatocellular carcinoma, ALI has been shown to be associated with immunotherapy response and prognosis [8]. ALI’s structure, which jointly evaluates inflammation and nutritional status, makes it potentially valuable for preoperative risk classification; however, its role in distinguishing benign from malignant conditions in gallbladder carcinoma has not yet been investigated.

In recent years, the CALLY index [(Albumin × Lymphocyte)/CRP] has gained attention for its strong prognostic value in hepatocellular carcinoma and pancreatic cancer, as it combines inflammation, immune response, and nutritional status into a single parameter [9]. According to literature reviews, the CALLY index has not been evaluated either prognostically or comparatively in gallbladder cancer, indicating a significant information gap.

This study aims to investigate whether ALI, CALLY, and other hematological inflammatory indices differ significantly between incidental GBC cases and age- and gender-matched benign cholecystitis patients. Thus, it aims to provide a new perspective on whether biological profiles indicating malignancy can be detected in the preoperative period.

## 2. Materials and Methods

This study uses a retrospective matched case–control design to evaluate cases of incidental gallbladder carcinoma (IGBC) found during histopathological examination after elective cholecystectomy. It includes all patients who had surgery between 2020 and 2025 at the General Surgery Clinic of Istanbul Basaksehir Cam and Sakura City Hospital.

The Clinical Research Ethics Committee of Istanbul Basaksehir Cam and Sakura City Hospital approved the study protocol (Number: 2025.11.416). Because the study is retrospective, the ethics committee waived the need for informed consent. All procedures followed the principles of the Declaration of Helsinki.

To minimize selection bias and control for potential confounders, matching was performed based on exact age and sex. Specifically, for each malignant case, control patients of the same age and sex were selected, without applying any age tolerance, resulting in a 1:4 matched case–control ratio. Control patients were randomly selected from the same surgical period using a computer-generated random sampling method from the eligible benign cholecystectomy cohort. Laboratory parameters were obtained from routine preoperative blood tests performed within 72 h prior to surgery. Inflammatory and immunonutritional indices were calculated using these preoperative values to ensure temporal consistency and avoid postoperative or disease-progression-related bias. Patients with missing laboratory data, active infection, hematological disorders, chronic inflammatory disease, or concurrent malignancy were excluded from index-based analyses. Patients who underwent surgery for acute cholecystitis were excluded. Acute cholecystitis was diagnosed according to the Tokyo Guidelines, based on the presence of compatible clinical findings, laboratory evidence of inflammation, and imaging criteria [10].

Researchers collected demographic data (age, sex, BMI), basic lab values (WBC, neutrophils, lymphocytes, monocytes, platelets, CRP, albumin, ALT, AST, GGT, total bilirubin, direct bilirubin), and pathological diagnoses from the electronic record system.

The inflammatory, immunonutritional, and hepatic function indices used in this study were calculated as defined in the literature. All formulas are presented in Table 1. To prevent errors caused by division by zero and missing data, the safe division function was applied in the calculations. Hematological indices such as NLR, derived neutrophil-to-lymphocyte ratio (dNLR; neutrophil/[WBC − neutrophil]), PLR, LMR, SII, CAR, ALI, and CALLY index were evaluated in our study.

### Statistical Analysis

All analyses were performed using R software (version 4.5.1; R Foundation for Statistical Computing, Vienna, Austria).

The distribution of continuous variables was assessed using the Shapiro–Wilk test. Normally distributed data were expressed as mean ± SD, and non-normally distributed data as median (IQR). Student’s *t*-test or Mann–Whitney U test was used to compare benign and malignant groups. Categorical variables were reported as frequency (%).

**Figure 1:**
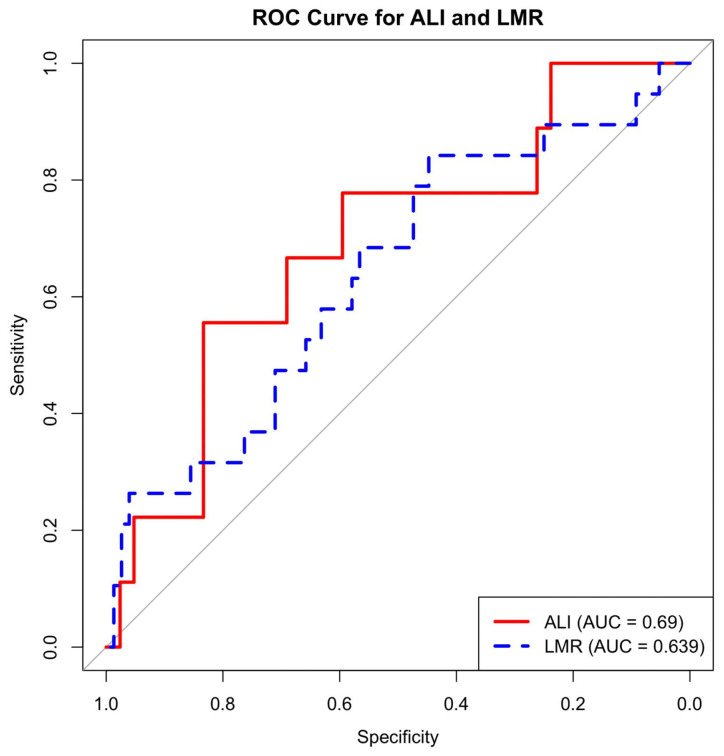
The ROC analysis demonstrates the discriminative performance of the Advanced Lung Cancer Inflammation Index (ALI) and the lymphocyte-to-monocyte ratio (LMR) in distinguishing incidental gallbladder carcinoma from benign gallbladder disease. ALI demonstrated moderate diagnostic accuracy with an AUC of 0.69, while LMR exhibited a moderate discriminative ability with an AUC of 0.639. The diagonal gray line indicates the reference line representing no discriminative capability.

The diagnostic performance of preoperative hematological and immunonutritional indices was evaluated using Receiver Operating Characteristic (ROC) analysis. For each index, AUC, optimal cut-off (Youden index), sensitivity, specificity, PPV, and NPV were calculated. ROC curves for ALI and LMR are presented in the main text as Figure 1, and detailed diagnostic criteria for all indices are presented in Supplementary Table S2.

In accordance with the matched case–control design, the conditional logistic regression (clogit) model was used to evaluate independent predictors associated with malignancy. To control for matching effects, a strata (set_id) structure was used at the set level. In accordance with the event-per-variable rule (EPV ≥ 10), only the two variables (ALI and LMR) with the highest discriminatory performance in the ROC analysis were included in the model. Unmatched univariate logistic regression models were not applied as they would compromise the methodological integrity of the matched design.

For the models, the adjusted odds ratio (OR), 95% confidence interval, likelihood ratio, Wald, and score test statistics were reported. Model performance was evaluated using the concordance statistic (c-index).

## 3. Results

A total of 95 cases were included in the study; 19 of these were classified as incidental gallbladder carcinoma, while 76 were classified as benign gallbladder disease. 19 1:4 matched sets were created based on age and gender matching, and all sets consisted of one malignant case and four benign cases.

No significant differences were found between the benign and malignant groups in basic demographic and biochemical parameters, including age, BMI, WBC, neutrophil, lymphocyte, monocyte, platelet, CRP, albumin, total bilirubin, direct bilirubin, ALT, AST, and GGT (all *p* > 0.10). The descriptive values for these variables are presented in Supplementary Table S1.

When derived inflammatory and nutritional indices were evaluated, none demonstrated statistically significant differences between the benign and malignant groups; however, lymphocyte-to-monocyte ratio (LMR) and advanced lung cancer inflammation index (ALI) exhibited borderline trends toward significance, representing the closest discriminatory inflammatory markers among the indices assessed. These comparisons are summarized in Table 2.

In ROC analysis based on raw ALI values, ALI demonstrated moderate discriminatory ability (AUC = 0.690). The optimal cut-off value was ALI = 497.38, corresponding to a sensitivity of 62.5% and a specificity of 83.7%. LMR showed a comparable but slightly lower discriminatory performance (AUC = 0.639). The AUC values for the remaining indices ranged from 0.53 to 0.60, indicating limited discriminatory power. The ROC curves for ALI and LMR are shown in Figure 1, and the cut-off values and diagnostic performance metrics for all indices are presented in Supplementary Table S2.

In the conditional logistic regression analysis within the matched case–control design, ALI showed an independent, inverse relationship with malignancy (adjusted OR = 0.997, 95% CI: 0.9937–1.0000). When ALI was rescaled per 100-unit increase, the adjusted odds ratio was 0.93 (95% CI: 0.57–1.39; *p* = 0.746), providing a clinically interpretable effect size. The likelihood ratio test (*p* = 0.04) and score test (*p* = 0.04) confirmed that ALI was a statistically significant predictor in the matched model. LMR did not reach statistical significance in the matched analysis (*p* = 0.12) and should therefore be considered a secondary, complementary marker, despite showing a consistent direction of association in ROC analysis. The regression results are presented in Table 3.

## 4. Discussion

Increased preoperative suspicion provides a critical advantage in the treatment algorithm. Ultrasonography is usually the first imaging modality used in chronic cholecystitis; however, the literature indicates that ultrasound has limited sensitivity for detecting GBC in its early stages [11]. If clinical or biochemical findings increase the likelihood of malignancy, additional cross-sectional imaging, such as contrast-enhanced computed tomography (CT), magnetic resonance imaging (MRI), or MRCP, may improve diagnostic accuracy [12]. Therefore, the presence of a biomarker indicating malignancy in the preoperative period can bring about a significant change in patient management.

IGBC is a malignancy reported in approximately 0.25–0.89% of cholecystectomy series, often unpredictable with preoperative imaging and detected by the pathology report. Tumor stage plays a decisive role in the decision for re-resection and long-term prognosis; while cholecystectomy is considered sufficient in T1a cases, additional hepatectomy and lymphadenectomy are recommended in T1b and higher cases. However, neither ultrasound nor cross-sectional imaging can reliably distinguish malignancy preoperatively in most cases [9,13]. Therefore, risk stratification for IGBC using inexpensive, accessible biomarkers derived from routine preoperative laboratory parameters has the potential to guide both surgical strategy and the intensity of pathological evaluation.

In this matched case–control study, we compared incidental gallbladder carcinoma (IGBC) cases found in routine cholecystectomy specimens with age- and sex-matched benign gallbladder disease cases to assess the predictive value of preoperative hematologic and inflammatory indices for malignancy. Of all the indices, only the Advanced Lung Cancer Inflammation Index (ALI) had the highest discriminatory power in the ROC analysis and showed an independent, inverse relationship with IGBC in the matched conditional logistic regression model. The lymphocyte-to-monocyte ratio (LMR) had limited diagnostic performance in the ROC analysis and did not reach statistical significance in the matched model. Classic inflammatory indices (NLR, PLR, SII, etc.) did not show significant differences between the benign and malignant groups.

The central role of chronic inflammation in the development of gallbladder carcinoma has long been recognized. Cholelithiasis, chronic cholecystitis, and bile stasis facilitate dysplastic–neoplastic transformation through mucosal damage, oxidative stress, bacterial toxins, and cytokine release [14]. This process is consistent with the classical framework of cancer-related inflammation; inflammatory cells and mediators contribute to the formation of a tumor microenvironment that supports proliferation, angiogenesis, invasion, and metastasis [15,16,17].

The fact that IGBC often has an asymptomatic course and the limited nature of imaging findings make peripheral blood indices reflecting systemic inflammation and immunonutrition clinically more attractive.

The most intensively studied hematological indices for gallbladder cancer in the literature are the neutrophil-to-lymphocyte ratio (NLR), platelet-to-lymphocyte ratio (PLR), monocyte-to-lymphocyte ratio (MLR), platelet count, and systemic immune–inflammation index (SII). Wu et al. compared the Glasgow Prognostic Score (GPS) and NLR in patients with resectable gallbladder carcinoma; they reported that both were associated with survival, but that GPS was superior as an independent prognostic marker [18]. Liu et al. demonstrated that high preoperative NLR and PLR values are associated with a poorer prognosis in patients with gallbladder carcinoma [19]. In the US Extrahepatic Biliary Malignancy Consortium data, Beal et al. reported that a high NLR was associated with poorer survival for both gallbladder cancer and cholangiocarcinoma [20]. Similarly, Liu et al. demonstrated that the combination of NLR and CA 19-9 predicts long-term outcomes in patients with gallbladder carcinoma who undergo curative surgery [21]. A recent systematic review and meta-analysis based on these individual studies has revealed that elevated levels of NLR, PLR, MLR, platelet count, and SII are significantly associated with poorer overall survival in gallbladder cancer [22].

In recent years, composite scores reflecting both systemic inflammation and nutritional status have emerged as stronger prognostic markers than classic single-parameter indices in many solid tumors [23,24]. The Advanced Lung Cancer Inflammation Index (ALI) is an immunonutrition index that combines BMI, serum albumin, and NLR and was first defined as a prognostic score in patients with metastatic non-small cell lung cancer [25]. Subsequently, low ALI levels have been shown to be associated with poorer survival in numerous gastrointestinal malignancies such as colorectal, gastric, hepatocellular carcinoma, pancreatic cancer, and cholangiocarcinoma [25]. However, data on ALI for gallbladder carcinoma are extremely limited, and no study focusing on preoperative benign–malignant differentiation in IGBC has been identified.

In this context, it is noteworthy that in our study, ALI emerged as the index with the best performance in distinguishing IGBC from benign gallbladder disease. The AUC value determined for ALI is 0.69, indicating moderate discriminatory power rather than being a perfect diagnostic test on its own. However, the relatively high specificity along with sensitivity, and particularly the negative predictive value of around 90%, suggests that it may help to categorize the likelihood of IGBC as “low” in low-risk populations. The fact that ALI maintained its inverse relationship with malignancy in the matched conditional logistic regression analysis and that statistical significance was preserved in the likelihood ratio and score tests supports that ALI remains an independent predictor even when controlling for basic confounding variables such as age and gender. Although LMR has higher sensitivity than ALI in ROC analysis, it did not reach significance in the matched model and should therefore be considered a secondary, complementary index.

All in all, the AUC value of ALI in our series remained below 0.7, making it clear that it is more rational for ALI to be integrated into a clinical and radiological risk score rather than being used alone as a “screening test” in clinical practice.

From the perspective of the clinical outcomes of our study, it is considered that a risk classification concept supported by ALI is worth testing in cases where there are no significant findings of malignancy in preoperative imaging, and cholecystectomy is planned due to symptomatic cholelithiasis. For example, in patients with suspicious imaging findings such as advanced age, thick-walled gallbladder, segmental wall irregularity, and a significantly low ALI value, advanced preoperative imaging, intraoperative frozen section planning, or surgical precautions to avoid gallbladder perforation may be prioritized [9,13]. Conversely, in cases where ALI is preserved with benign imaging findings, the likelihood of IGBC is low; nevertheless, it should be remembered that careful histopathological examination of all cholecystectomy specimens is essential. In this context, low ALI values may be considered as supportive preoperative risk stratification signals that prompt closer attention to radiologic red flags and consideration of additional imaging or hepatobiliary consultation when clinically appropriate.

This study has several important strengths. First, a matched case–control design was used, and each IGBC case was matched with four benign cases in terms of age and gender, thereby minimizing the effect of these two important confounding variables. Second, the inclusion of cases that underwent surgery with a “benign preliminary diagnosis” and were found to have incidental malignancy represents the real problem in clinical practice, namely, patients whose malignancy cannot be predicted preoperatively. Third, the systematic evaluation of numerous inflammatory and immunonutrition indices in the analysis clarified the position of ALI among other scores. Finally, the use of matched conditional logistic regression and limiting the number of variables to meet the events per variable recommendations reduced the risk of model overfitting [26].

However, our findings should be interpreted within certain important limitations. The study is retrospective and single-center, and the number of IGBC cases is relatively limited; this has both restricted the number of predictive variables and led to relatively wide confidence intervals. The odds ratio identified in the conditional logistic regression model for ALI corresponds to a one-unit increase, which may require rescaling to clinically meaningful intervals (e.g., specific percentiles of ALI) for a more meaningful interpretation in practice. The cut-off values determined by ROC analysis are specific to our dataset and should not be directly transferred to different populations without external validation. Furthermore, tumor markers, radiological risk scores, or histopathological parameters were not integrated into the model in this study; therefore, the performance of combined models incorporating ALI with these variables is unknown. Finally, laboratory parameters were obtained from a single preoperative time point; the lack of evaluation of dynamic changes or postoperative evolution is another limitation. Because incidental gallbladder carcinoma is rare, the limited number of malignant cases may have reduced statistical power and increased the risk of type II error, particularly for secondary indices such as LMR.

## 5. Conclusions

In this matched case–control study, preoperative hematological and immunonutritional indices were evaluated in incidental gallbladder carcinoma cases; among these indices, only the ALI (Advanced Lung Cancer Inflammation Index) showed an independent, inverse relationship with malignancy in both diagnostic performance and matched conditional logistic regression analyses. LMR (Lymphocyte-to-Monocyte Ratio) had moderate discriminatory power in ROC analysis but did not reach statistical significance in the matched model. The study findings suggest that ALI may be a potential biomarker for predicting incidental malignancy, but it is not sufficient to replace preoperative clinical and radiological evaluation alone.

## Figures and Tables

**Table 1:** Definitions and Mathematical Formulas of Inflammatory and Nutritional Indices Used in This Study.

Index	Formula	Description
NLR (Neutrophil-to-Lymphocyte Ratio)	Neutrophil/Lymphocyte	Marker of systemic inflammation.
dNLR (Derived Neutrophil-to-Lymphocyte Ratio)	Neutrophil/(WBC − Neutrophil)	Modified NLR incorporating total WBC count.
PLR (Platelet-to-Lymphocyte Ratio)	Platelet/Lymphocyte	Reflects thrombocytosis and lymphopenia in inflammatory states.
LMR (Lymphocyte-to-Monocyte Ratio)	Lymphocyte/Monocyte	Indicator of immune status and monocyte-related tumor activity.
SII (Systemic Immune–Inflammation Index)	(Platelet × Neutrophil)/Lymphocyte	Composite marker integrating neutrophils, platelets, and lymphocytes.
CAR (CRP-to-Albumin Ratio)	CRP/Albumin	Composite index reflecting inflammation and nutritional reserve.
ALI (Advanced Lung Cancer Inflammation Index)	(BMI × Albumin)/NLR	Composite score incorporating BMI, albumin, and inflammation.
CALLY Index	(Albumin × Lymphocyte)/CRP	Reflects immunonutrition adjusted for systemic inflammation.

Legend: All indices were computed from preoperative laboratory variables. Mathematical formulas are presented below. Reference citations will be added after final selection of supporting literature.

**Table 2:** Baseline Laboratory and Derived Indices in Benign vs. Malignant Gallbladder Cases.

Variable	Benign Median (IQR)	Malignant Median (IQR)	*p*-Value
Age (years)	60 (50–65)	60 (50.5–65)	0.9888
BMI (kg/m^2^)	31.20 (27.74–34.04)	28.73 (28.40–33.20)	0.4363
WBC (×10^9^/L)	7.83 (6.21–9.16)	7.20 (6.26–8.64)	0.7202
Platelet (×10^9^/L)	266.5 (233.8–307.3)	264.0 (220–312.5)	0.7517
Neutrophils (×10^9^/L)	4.40 (3.65–5.37)	4.26 (3.55–5.41)	0.8088
Lymphocytes (×10^9^/L)	2.25 (1.75–2.82)	1.95 (1.62–2.54)	0.1656
Monocytes (×10^9^/L)	0.55 (0.45–0.69)	0.58 (0.53–0.75)	0.2227
CRP (mg/L)	3.75 (2.10–8.18)	4.80 (2.85–16.20)	0.2211
Albumin (g/L)	42 (40.25–45.75)	41 (39.5–43.5)	0.3459
Total bilirubin (mg/dL)	0.385 (0.288–0.513)	0.410 (0.265–0.580)	0.6787
Direct bilirubin (mg/dL)	0.155 (0.110–0.208)	0.165 (0.123–0.285)	0.4334
ALT (U/L)	18 (12–25)	16 (11.5–24.5)	0.7978
AST (U/L)	20 (15–25)	20 (15–24.5)	0.9073
GGT (U/L)	25 (18.5–47.8)	23 (14–30.5)	0.2877
NLR	1.98 (1.44–2.51)	2.21 (1.67–2.72)	0.3039
dNLR	1.43 (1.13–1.82)	1.54 (1.18–1.85)	0.6754
PLR	119.9 (92.8–158.7)	122.3 (102.9–169.5)	0.5119
LMR	3.99 (3.30–5.05)	3.54 (2.77–4.05)	0.0621
SII	521.0 (417.4–669.2)	549.5 (418.1–878.5)	0.5484
ALI	730.3 (548.5–894.9)	497.4 (493.9–668.8)	0.0773
CALLY index	0.00248 (0.00116–0.00449)	0.00188 (0.000466–0.00337)	0.1715

Legend: Benign group: *n* = 76; Malignant group: *n* = 19.

**Table 3:** Conditional Logistic Regression (Matched Analysis).

Variable	Adjusted OR	95% CI	*p*-Value (LR Test)
ALI	0.997	0.9937–1.0000	0.04
LMR	0.737	0.4998–1.087	0.08

Legend: Matched conditional logistic regression stratified by set_id. *p*-values from likelihood ratio test.

## Data Availability

The data presented in this study are available on reasonable request from the corresponding author.

## Supplementary Materials

The following supporting information can be downloaded at: https://www.mdpi.com/article/10.3390/medicina62020269/s1, Table S1: Baseline laboratory values in benign vs malignant cases; Table S2: Inflammatory and Nutritional Indices; Table S3: ROC Performance and Optimal Cut-off Values.
