# Supplementary material for: Preoperative Advanced Lung Cancer Inflammation Index as a Potential Marker for Incidental Gallbladder Carcinoma: A Matched Case–Control Study
Source: Medicina (Kaunas). 2026 Jan 27;62(2):269. doi: 10.3390/medicina62020269 (PMC12942496; doi:10.3390/medicina62020269)
Supplement: Supplementary file 1 [file medicina-62-00269-s001.zip › medicina-4101334-supplementary.pdf]

Supplementary Table S1. Baseline laboratory values in benign vs malignant cases

**Variable**	**Benign Median (IQR)**	**Malignant Median (IQR)**
**Age (years)**	60 (50–65)	60 (50.5–65)
**BMI (kg/m^2)**	31.20 (27.74–34.04)	28.73 (28.40–33.20)
**WBC (×10^9/L)**	7.83 (6.21–9.16)	7.20 (6.26–8.64)
**Platelet (×10^9/L)**	266.5 (233.8–307.3)	264.0 (220–312.5)
**Neutrophils (×10^9/L)**	4.40 (3.65–5.37)	4.26 (3.55–5.41)
**Lymphocytes (×10^9/L)**	2.25 (1.75–2.82)	1.95 (1.62–2.54)
**Monocytes (×10^9/L)**	0.55 (0.45–0.69)	0.58 (0.53–0.75)
**CRP (mg/L)**	3.75 (2.10–8.18)	4.80 (2.85–16.20)
**Albumin (g/L)**	42.0 (40.25–45.75)	41.0 (39.5–43.5)
**Total bilirubin (mg/dL)**	0.385 (0.288–0.513)	0.410 (0.265–0.580)
**Direct bilirubin (mg/dL)**	0.155 (0.110–0.208)	0.165 (0.123–0.285)
**ALT (U/L)**	18 (12–25)	16 (11.5–24.5)
**AST (U/L)**	20 (15–25)	20 (15–24.5)
**GGT (U/L)**	25 (18.5–47.8)	23 (14–30.5)

Supplementary Table S2. Inflammatory and Nutritional Indices

**Variable**	**Benign Median (IQR)**	**Malignant Median (IQR)**
**NLR**	1.98 (1.44–2.51)	2.21 (1.67–2.72)
**dNLR**	1.43 (1.13–1.82)	1.54 (1.18–1.85)
**PLR**	119.9 (92.8–158.7)	122.3 (102.9–169.5)
**LMR**	3.99 (3.30–5.05)	3.54 (2.77–4.05)
**MLR**	0.25 (0.20–0.30)	0.28 (0.25–0.36)
**SII**	521.0 (417.4–669.2)	549.5 (418.1–878.5)
**CAR**	0.089 (0.053–0.190)	0.128 (0.063–0.387)
**ALI**	730.3 (548.5–894.9)	497.4 (493.9–668.8)
**CALLY index**	0.00248 (0.00116–0.00449)	0.00188 (0.000466–0.00337)

Legend:
Diagnostic performance metrics were calculated using ROC curves based on logistic regression–derived predicted probabilities. Optimal threshold represents the Youden index (sensitivity + specificity – 1).

Supplementary Table S3. ROC Performance and Optimal Cut-off Values

**Index**	**AUC**	**Opt□mal Cut-off (Youden)**	**Sens□t□v□ty**	**Spec□f□c□ty**	**PPV**	**NPV**
**ALI**	**0.690**	**0.253**	**0.556**	**0.833**	0.417	**0.897**
**LMR**	0.639	0.194	**0.842**	0.447	0.276	**0.919**
**CALLY**	0.602	0.237	0.316	**0.932**	0.545	0.841
**NLR**	0.577	0.198	0.579	0.618	0.275	0.855
**PLR**	0.549	~0.18	**0.895**	0.263	0.233	**0.909**
**SII**	0.545	0.201	0.474	0.737	0.310	0.848
**dNLR**	0.532	0.199	**0.947**	0.237	0.237	**0.947**

Legend: *Diagnostic performance metrics were calculated using ROC curves based on logistic regression–derived predicted probabilities. Optimal threshold represents the Youden index (sensitivity + specificity – 1).*
